# Comparison of Antibacterial Activities of Korean Pine (*Pinus densiflora*) Needle Steam Distillation Extract on *Escherichia coli* and *Staphylococcus aureus* Focusing on Membrane Fluidity and Genes Involved in Membrane Lipids and Stress

**DOI:** 10.3390/molecules29010165

**Published:** 2023-12-27

**Authors:** Ya Zhang, Woo-Kyung Chung, Su-Hyun Moon, Jeoung-Gyu Lee, Ae-Son Om

**Affiliations:** Department of Food and Nutrition, Hanyang University, Seoul 04736, Republic of Korea; zhangya190726@163.com (Y.Z.); entksv10@naver.com (W.-K.C.); anstngus9205@naver.com (S.-H.M.); angelhb2@navr.com (J.-G.L.)

**Keywords:** antibacterial activity, mechanism, *Pinus densiflora*, cell membrane, fatty acid genes

## Abstract

The antibacterial activity and mechanism of *Pinus densiflora* extracts against *Escherichia coli* and *Staphylococcus aureus* were investigated. The growth inhibition tests of paper diffusion and optical density exhibited that the extracts have potent antibacterial potentials against foodborne pathogens. The measurement of membrane fluidity by fluorescence polarization has indicated that one of the antibacterial mechanisms involves the disruption of membrane integrity resulting in an increase in the membrane fluidity in both of *E. coli* and *S. aureus*. The alteration of fatty acid composition was accompanied by the disturbance of membranes thus shifting the proportion of saturated verses unsaturated fatty acids or trans fatty acids from 1.27:1 to 1.35:1 in *E. coli* and 1.47:1 to 2.31:1 in *S. aureus*, most likely to compensate for the increased membrane fluidity by means of a higher proportion of saturated fatty acids which is known to render rigidity in membranes. Realtime q-PCR (polymerase chain reaction) analysis of fatty acid synthetic genes and bacterial stress genes revealed that there was minimal influence of *P. densiflora* extracts on fatty acid genes except for *fab I* and the stress *rpos* in *E. coli*, and relatively greater impact on fatty acid genes and the stress *sigB* in *S. aureus*.

## 1. Introduction

*Pinus densiflora* is an evergreen needle-leaf tree indigenous to East Asia, and its parts that are mainly used in medicine are the leaves, bark, and cones [1]. These parts have been used in traditional, folk, or official medicines for remedies against various diseases and disorders because they contain medicinal components showing antioxidant, antimicrobial, antitumor, and antimutagenic properties. According to previous studies, the main components of *P. densiflora* are α-pinene, β-pinene, myrcene, and limonene [2,3]. Given the increasing interest in natural products with antioxidant and antimicrobial effects, research on pine needle trees as the effective antimicrobial agent is being conducted [4,5,6,7]. Specifically, the antimicrobial activities of flavonoids found richly in *P. densiflora* have been drawing more attention [8,9]. Recently, the possibilities of developing *P. densiflora* extracts as naturally occurring antibacterial agents targeting food, cosmetics, and the agricultural industry have emerged greatly. Generally, consumers have shown much preference towards non-artificial natural products with safer, fresher, and healthier components rendering a high demand for natural and safe preservation technologies. Therefore, research on the nature of natural products and their mechanisms of action against microorganisms would be considered urgent matters.

It has been extensively shown that *P. densiflora* inhibited the growth of foodborne pathogenic organisms such as *Staphylococcus aureus*, *Escherichia coli*, and *Salmonella typhimurium*. Although *P. densiflora* itself or its components such as Shikimic acid have been investigated and applied as antibacterial agents, there is very limited extensive research on the modes of action along with molecular mechanisms. Most of the antibacterial agents exert effects through primary binding to bacterial membranes and then render them permeable through formation of pores. *P. densiflora* possesses an additional desirable property of selectiveness not harming host cells. Most likely, these kinds of observations might be due to the fact that the microbial membranes have negatively charged phosphatidylglycerol and cardiolipin on their surface, whereas in eukaryotic cell membrane anionic lipids are found to be segregated to the inner leaflet which exclude as direct targets of *P. densiflora*. In reconstituted membranes, flavonoids from various plant sources interact more strongly against the anionic state membrane compared to the cationic state [10,11,12]. Several antibacterial agents were found to attach directly to the lipid structure of bacterial membranes resulting in an increased membrane fluidity, and further increasing pores and forming micelles [13,14,15]. However, bacteria are capable of adapting their membrane structures to survive from these detrimental effects by the alteration of the membrane fatty acid profiles [16,17,18]. These modifications of lipid profiles may exert maintaining membrane integrity as well as functionality or reducing interaction between bacteria and antimicrobial agents. Other than the direct destruction of the bacterial cell membranes or cell walls, antibacterial agents have been found to prevent or impede the development of microorganisms through the interaction with bacterial genes related to bacterial survival [19,20].

Bacteria can develop the capacity to resist by producing factors called stress-response alternative sigma factors *rpoS* and *sigB* [21,22,23]. The effect of modulation of these two factors by bacteria under *P. densiflora* treatment can provide the insight of antimicrobial mechanisms of plant origin antibacterial products. In this study, antioxidant, antibacterial activities, and the antibacterial mechanisms focusing on the influence of cell membrane integrity and genes involved in membrane lipid composition and alternative stress factors were investigated. First, the total polyphenol content (TPC) and total flavonoid content (TFC) of *P. densiflora* extracts were measured, the antioxidant effects were assessed, and then the antibacterial activities for five foodborne pathogens were scrutinized. It was found that Gram-negative bacteria were more sensitive to the extract tested than Gram-positive bacteria. In order to elucidate the differences in sensitivities against Gram-positive and Gram-negative bacteria, the comparison of antibacterial activities against Gram-negative *E. coli* and Gram-positive *Staphylococcus aureus* focusing on differing patterns of membrane integrity and representative fatty acids genes crucial to the retarded growth of bacteria by the pine needle extracts was studied. Also, the changes in gene expression of general stress genes of these two bacteria as a result of exposure to *P. densiflora* was investigated.

## 2. Results

### 2.1. Antioxidant Effects of Steam Distilled Pine Needle Extracts

**Figure 1:**
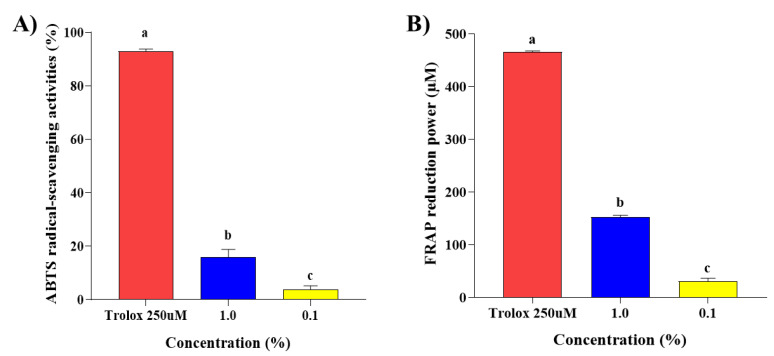
Antioxidant effects of steam distilled pine needle (*Pinus densiflora*) extracts. Antioxidant effects were determined by (**A**) ABTS radical scavenging assay and (**B**) FRAP assay. SDPNE was diluted with 95% ethyl alcohol. Values are presented as mean ± standard deviation. Statistically significant differences (*p* < 0.05) between groups were determined by one-way ANOVA and Duncan’s multiple range test. Different letters indicate statistically significant differences. ABTS, 2,2′-azino-bis (3-ethylbenzothiazoline-6-sulfonic acid); FRAP, ferric reducing antioxidant power.

TFC and TPC in Steam Distilled Pine Needle Extract (SDPNE) were measured (Table 1). TPC of SDPNE was 3.61 ± 0.002 mg GAE/g and TFC was 24.97 ± 1.55 mg QE/100 g. TPC of SDPNE was calculated as mg gallic acid equivalents per gram (mg GAE/g) and TFC of SDPNE was expressed as mg quercetin equivalents per 100 g (mg QE/100 g). The antioxidant effect of SDPNE at the concentration of 1 and 0.1% was analyzed by ABTS and FRAP assays. ABTS radical scavenging activity of SDPNE was shown as 15.95 ± 2.87 and 3.82 ± 1.39% at 1 and 0.1%, respectively (Figure 1A). Also, the result of the FRAP assay indicated 152.77 ± 3.26 μM FeSO_4_/mL for 1% and 30.97 ± 5.87 μM FeSO_4_/mL for 0.1%, respectively (Figure 1B). Additionally, 1% SDPNE showed the highest antioxidant activities of 17.15 ± 3.23% and 32.77 ± 0.81% in ABTS and FRAP assays compared to 250 μM Trolox, indicating SDPNE possesses relatively high antioxidant capacity.

### 2.2. Antibacterial Activity of Steam Distilled Pine Needle Extract

**Figure 2:**
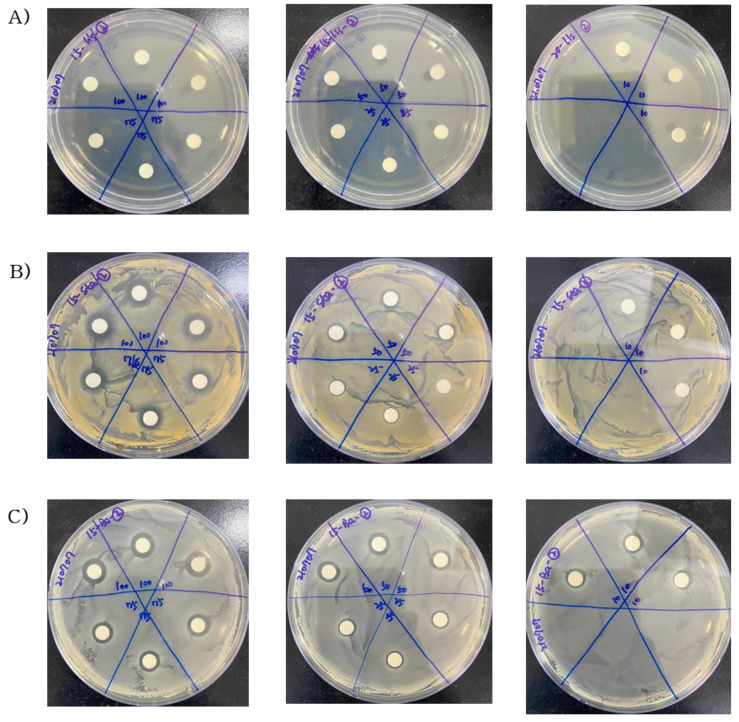
The effect of the antibacterial activity of steam distilled pine needle (*Pinus densiflora*) extracts on Gram-positive bacteria (**A**) *Listeria monocytogenes*, (**B**) *Staphylococcus aureus*, and (**C**) *Bacillus subtilis* for 24 h. The concentration of SDPNE were 100, 75, 50, 25, and 10%, respectively.

**Figure 3:**
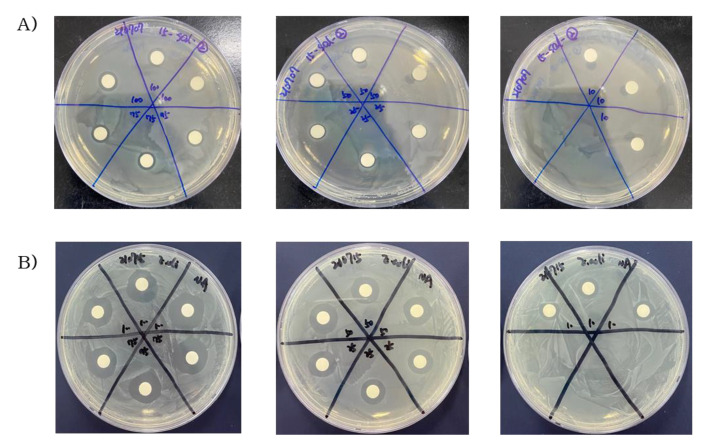
The effect of the antibacterial activity of steam distilled pine needle (*Pinus densiflora*) extracts on Gram-negative bacteria (**A**) *Salmonella enteritidis* and (**B**) *Escherichia coli* for 24 h. The concentration of SDPNE were 100, 75, 50, 25, and 10%.

**Figure 4:**
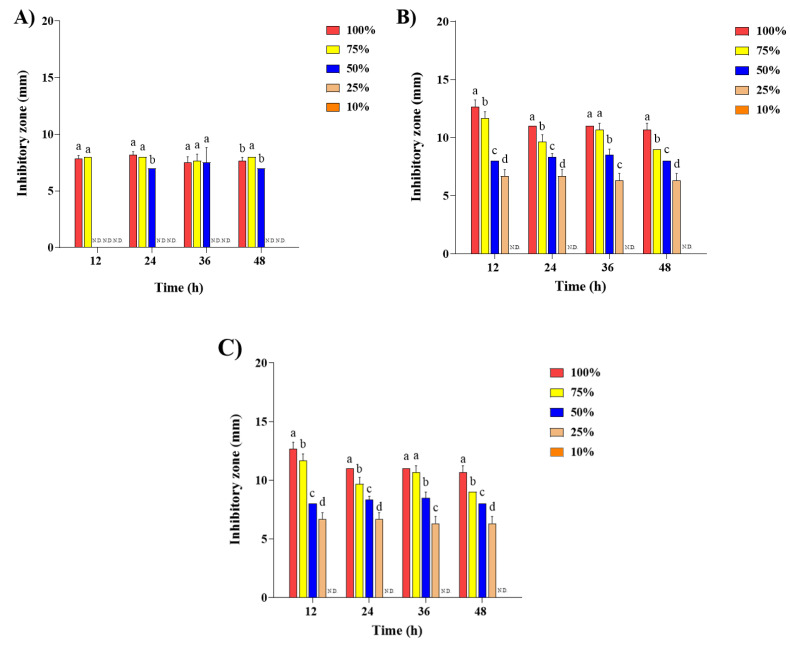
The effect of the antibacterial activity of steam distilled pine needle (*Pinus densiflora*) extracts on Gram-positive bacteria (**A**) *Listeria monocytogenes*, (**B**) *Staphylococcus aureus*, and (**C**) *Bacillus subtilis* for 48 h. Significant differences between groups were analyzed by one-way ANOVA (Duncan’s multiple range test; *p* < 0.05). Different letters are indicated as significant differences.

**Figure 5:**
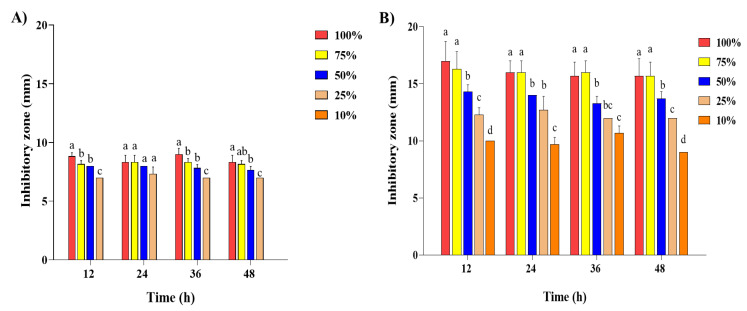
The effect of the antibacterial activity of steam distilled pine needle (*Pinus densiflora*) extracts on Gram-negative bacteria (**A**) *Salmonella enteritidis* and (**B**) *Escherichia coli* for 48 h. Significant differences between groups were analyzed by one-way ANOVA (Duncan’s multiple range test; *p* < 0.05). Different letters are indicated as significant differences.

The effect of the antibacterial activity of SDPNE on foodborne bacteria was analyzed by the paper disk method. Gram-positive (*L. monocytogenes*, *S. aureus*, and *B. subtilis*) and Gram-negative bacteria (*S. enteritidis* and *E. coli*) were treated for 12, 24, and 48 h at 100, 75, 50, 25, and 10% of SDPNE. The results showed sustained antibacterial effects (Figure 2, Figure 3, Figure 4 and Figure 5). Figure 2A and Figure 4A showed that at 12 h, only 100% (7.8 ± 0.3 mm) and 75% (8.0 ± 0.0 mm) SDPNE inhibited the growth of *L. monocytogenes*. With the continuous action of SDPNE, 50% SDPNE also showed an inhibitory effect on *L. monocytogenes* at 24 h (7.0 ± 0.0 mm). At 12 h, the highest antibacterial activity occurred in *S. aureus* (12.6 ± 0.6 mm), when compared to other Gram-positive bacteria. (Figure 4B). However, there was no inhibitory zone at 10% SDPNE (≤6 mm) exposure in *S. aureus* and *S. enteritidis* (Figure 2B and Figure 3A). For *B. subtilis*, all concentrations of SDPNE produced an inhibitory zone (≥6 mm) (Figure 4C). In Figure 3B and Figure 5B, a strong inhibitory effect on *E. coli* for 12 h after treated with 100% (17.0 ± 1.7 mm) and 10% (10.0 ± 0.0 mm) of SDPNE, respectively.

**Figure 6:**
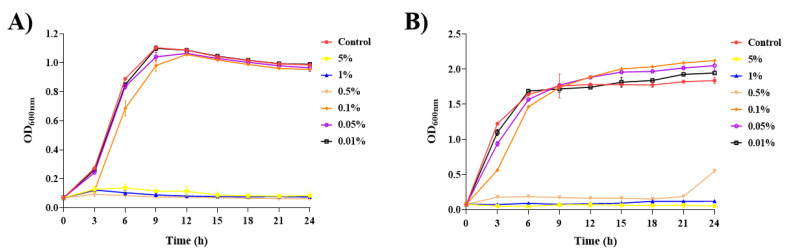
The effect of steam distilled pine needle (*Pinus densiflora*) extracts on bacterial cell growth of (**A**) *Escherichia coli* and (**B**) *Staphylococcus aureus.* The optical density measured the growth at 600 nm.

The effect of SDPNE on the growth rate of *E. coli* and *S. aureus* was determined by measuring optical density at 600 nm (OD_600_) (Figure 6). The growth of *E. coli* increased in a dose-dependent manner of SDPNE (0.01%, 1.10 ± 0.02 nm; 0.05%, 1.04 ± 0.03 nm, and 0.1%, 0.98 ± 0.04 nm) at 9 h (Figure 6A). *E. coli* did not show significant growth after being treated with 0.1% of SDPNE for 3 h (0.12 ± 0.00 nm). The growth of *E. coli* exposed to 0.01, 0.05, and 0.1% SDPNE for 12 h was consistent with the control group (without SDPNE) (OD_600_ ≈ 1.08 nm). *E. coli* did not grow within 24 h when exposed to higher concentrations ranging from 0.5 to 5%.

As shown in Figure 6B, the OD_600_ of *S. aureus* increased with the decreasing concentrations (0.01, 0.05, and 0.1%) of SDPNE over 9 h and reached the stationary phase at 9 h. The OD values were 1.07 ± 0.05 nm, 1.77 ± 0.02 nm, and 1.74 ± 0.02 nm, respectively, when *S. aureus* was exposed to 0.01, 0.05, and 0.1% SDPNE at 3 h. In 5% SDPNE, OD_600_ did not change within 21 h, but increased to 0.55 ± 0.00 nm at 24 h. The OD_600_ value did not change in *S. aureus* within 24 h at 1% and 5% SDPNE.

**Figure 7:**
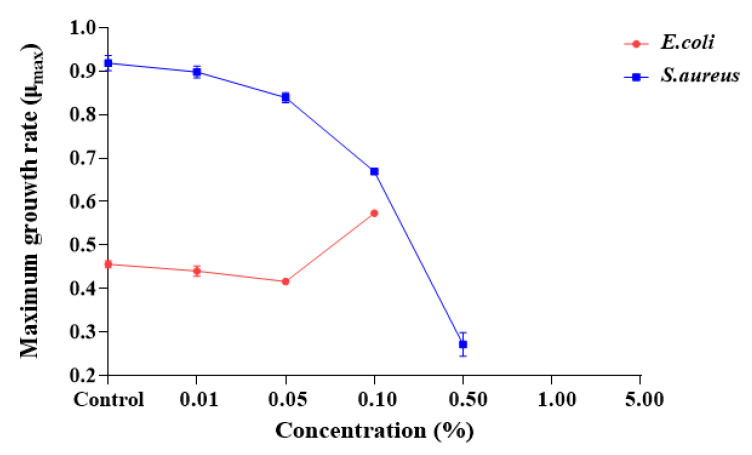
The effect of pine needle (*Pinus densiflora*) steam distillation extract on the highest maximum growth rate (μ_max_) of *Escherichia coli* and *Staphylococcus aureus* maximum growth rate (μ_max_) measured by the optical density at 600 nm.

As shown in Figure 7, the maximum growth rate (μ_max_) of *E. coli* and *S. aureus* was measured after exposure to different concentrations of SDPNE. The μ_max_ of *E. coli* and *S. aureus* in the control group were 0.46 ± 0.01 and 0.93 ± 0.00, respectively. The μ_max_ of *E. coli* was over 0.40 at 0.01, 0.05, and 0.1%. The highest maximum growth rate was 0.57 ± 0.00 when exposed to 0.1% of SDPNE for 9 h. For *S. aureus*, the μ_max_ were 0.89 ± 0.01 for 0.01% and 0.84 ± 0.01 for 0.05%, respectively.

### 2.3. Membrane Fluidity Changes under SDPNE Treatments

**Figure 8:**
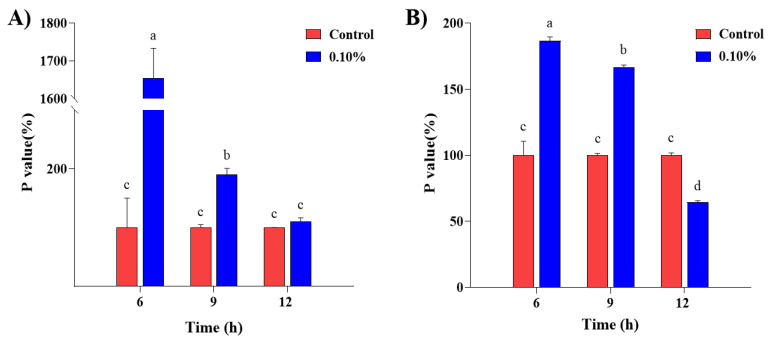
Correlation of fluorescence polarization of (**A**) *Escherichia coli* and (**B**) *Staphylococcus aureus* after exposure to steam distilled pine needle extracts at a concentration of 0.1% at 6, 9, and 12 h. Significant differences between groups were analyzed by one-way ANOVA (Duncan’s multiple range test; *p* < 0.05). Different letters are indicated as significant differences.

The effect of fluorescence polarization of *E. coli* and *S. aureus* after being cultivated at 0.1% of SDPNE was shown in Figure 8. The polarization (P) values indicated that the membrane fluidity of *E. coli* and *S. aureus* were decreased in the presence of SDPNE. The *p* values of these two kinds of bacteria decreased in a time-dependent manner from 6 to 12 h when exposed to a concentration of 0.1% SDPNE. Results of membrane fluidity of *E. coli* exposed to SDPNE at 0.1% were shown in Figure 8A. The highest *p* value was 1655.24 ± 78.53% at 6 h, but the *p* value of 12 h was 110.37 ± 6.39%, which was of no statistical significance compared to the control group (*p* > 0.05). Similarly, a significant decrease in *p* values (from 6 to 12 h) was observed when *S. aureus* was exposed to 0.1% SDPNE (Figure 8B). At 12 h, the P value of *S. aureus* was significantly decreased (64.50 ± 1.17%) (*p* < 0.05).

### 2.4. Membrane Fatty Acid Compositions of Escherichia coli and Staphylococcus aureus with SDPNE

Membrane FA composition of *E. coli* and *S. aureus* were measured at 6, 9, and 12 h of 0.1% SDPNE exposure compared to the control. The total 12 FAs were identified and quantified in *E. coli* (lauric acid [C12:0], tetradecanoic acid [C14:0], pentadecanoic acid [C15:0], palmitic acid [C16:0], hexadecanoic acid [C17:0], stearic acid [C18:0], pamitoleic acid [C16:1], cis-11-eicosenoic acid [C20:1], oleic acid [cis-C18:1], elaidic acid [trans-C18:1], linoleic acid [cis-C18:2], and linolelaidic acid [trans-C18:2]). FAs were divided into saturated fatty acids (SFAs) and unsaturated fatty acids (UFAs). The content of UFAs in *E. coli* decreased from 42.63% at 6 h to 38.81% at 12 h, while the content of SFAs increased from 57.37% at 6 h to 61.19% at 12 h (Table 2). At 12 h, the proportion of C20:1 in the control and 0.1% of SDPNE were 0.71 and 0.57%, respectively (Table 3). The *trans*-C18:1n-9 was observed as 2.3% at 6 h and 2.07% at 9 h. The *cis*-C18:2n-6 was not detected at 6 h.

Correspondingly, a total of 10 FAs were identified in the membrane of *S. aureus*. The content of UFAs in *S. aureus* increased from 30.23% at 6 h to 40.79% at 12 h, and the content of SFAs increased from 69.77% at 6 h to 59.21% at 12 h, with a 0.1% concentration of SDPNE exposure (Table 2). The levels of C16:0, C18:0, and C20:0 at 0.1% SDPNE were consistently higher than the control group (Table 3). The *trans*-C18:1n-9 increased from 13.95% at 6 h to 23.03% at 12 h.

### 2.5. The Transcription of Fatty Acid Biosynthesis Gene

**Figure 9:**
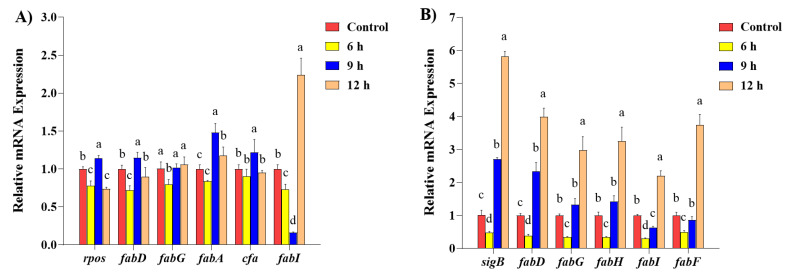
The expression levels of FA biosynthesis genes and alternative sigma factors (**A**) *Escherichia coli* and (**B**) *Staphylococcus aureus.* Significant differences between groups were analyzed by one-way ANOVA (Duncan’s multiple range test; *p* < 0.05). Different letters are indicated as significant differences.

The expression levels of FA biosynthesis genes and alternative sigma factors in *E. coli* and *S. aureus* after 0.1% SDPNE exposure at 6, 9, and 12 h are shown in Figure 9. A significant decrease in the transcription levels of FA biosynthesis genes was observed, which for *E. coli* saw the presence of 0.1% SDPNE for 6 h (*fabD*, 0.72 ± 0.06; *fabG*, 0.80 ± 0.06; *fabA*, 0.84 ± 0.01, and *fabI*, 0.73 ± 0.07) compared to the control (*p* < 0.05) (Figure 9A). For 9 h, the expression level of *fabI* was 0.16 ± 0.01 and all the other FA biosynthesis genes were upregulated. For 12 h, the expression level of *fabI* significantly increased to 2.24 ± 0.22-fold compared to the control (*p* < 0.05), but there was no statistical significance in *fabD*, *fabG*, and *cfa* when compared to the control group. The expression levels of general stress sigma factors *rpos* in *E. coli* decreased to 0.78 ± 0.06 for 6 h, 1.14 ± 0.04 for 9 h, and 0.74 ± 0.02 for 12 h. The levels of FA biosynthesis genes which *S. aureus* significantly decreased for 6 h (*fabD*, 0.39 ± 0.04; *fabG*, 0.34 ± 0.02; *fabH*, 0.35 ± 0.01; *fabI*, 0.30 ± 0.02; and *fabF*, 0.49 ± 0.05) compared to the control (*p* < 0.05) (Figure 9B). However, for 9 h, the transcription levels of *fabD* (2.33 ± 0.28), *fabG* (1.33 ± 0.18), and *fabH* (1.43 ± 0.17) were increased compared to the control. The *fabI* were 0.35 ± 0.01 at 6 h and 0.62 ± 0.05 at 9 h. For 12 h, the levels of *fabD* (3.99 ± 0.26), *fabG* (2.98 ± 0.41), *fabH* (3.25 ± 0.42), *fabI* (2.20 ± 0.15), and *fabF* (3.75 ± 0.31) were significant increases (*p* < 0.05). The expression levels of general stress sigma factors *sigB* in *S. aureus* were expressed at 6, 9, and 12 h after treatment with 0.1% SDPNE (Figure 9B). Similar to FA biosynthesis genes, *sigB* significantly increased in a time-dependent manner (*p* < 0.05), showing increasing levels of 0.48 ± 0.04, 2.70 ± 0.05, and 5.83 ± 0.15 at 6, 9, and 12 h, respectively.

## 3. Discussion

Pine needle extract is an efficient antimicrobial agent with a broad antibacterial spectrum [4,24]. Demonstrating antibacterial activities of pine needle extracts is of special concern for combating Gram-positive and Gram-negative foodborne and aquatic pathogens [25]. Especially, antimicrobial resistance threatens human health, and new types of antimicrobial agents are desperately needed. Prior to determination of antibacterial activities of *P. densiflora*, the phenol and flavonoid contents were determined and found in considerably higher amounts (3.61 ± 0.002 mg GAE/g, 24.97 ± 1.55 mgQE/100 g, respectively) compared to other kinds of flavonoid rich plants such as those in *Quercus, Rubus coreanus Miq.*, and *Rubia cordifolia* [26,27,28]. Flavonoids are a group of compounds that have more than two hydroxyl radicals attached to benzene rings possessing antioxidant and anti-inflammatory effects [29] often targeting degenerative diseases such as atherosclerosis, diabetes, and cancers, as well as bacterial contamination. Pine needles contain rich flavonoid components and, furthermore, flavonols containing prenylated side chains may have advantages over other kinds of plant extracts [8]. Antioxidant activities of *P. densiflora* extracts were compared to standard compound ascorbic acid and comparable antioxidant activities were observed. HPLC analysis of pine needle hot water extracts has shown that the major component exhibiting antioxidant activities is proanthocyanidine(s), a group of polyphenol bioflavonoids with a concentration of 30.54 mg/g extract [30].

The in vitro antibacterial activities of pine needle extracts were investigated by applying the paper diffusion method. The inhibitory activities of *P. densiflora* extracts on the Gram-negative bacteria (*E. coli* ATCC 25922) and the Gram-positive bacteria (*Staphylococcus aureus* ATCC 6538) were 17.0 ± 1.7 mm (*E. coli*) and 12.6 ± 0.6 mm (*S. aureus*), indicating the extract of this study was more effective in controlling Gram-negative bacteria than Gram-positive bacteria. Also, the growth inhibition rates as determined by optical densities measurement confirmed that *P. densiflora* extracts (0.5%) were more effective in controlling Gram-negative *E. coli* than Gram-positive *S. aureus*.

Membrane fluidity or integrity of *E. coli* and *S. aureus* with *P. densiflora* extracts were examined by fluorescence propidium iodide probe polarization analysis measuring the intensity of hydrophobic dyes embedded in fatty acid bilayers. Proper fluidity is detrimental to the cytoplasmic membrane of bacteria to maintain normal cellular functions, such as cell division, diffusion, and pumping ions [31]. In general, the membrane fluidity is higher in the logarithmic phase and lower in the stationary phase [32]. Thus, the membrane fluidity could represent the condition of the bacterial cells or organs in other species. Membrane fluidity was increased by treating with the extracts in both *E. coli* and *S. aureus* for up to 6 h of incubation suggesting that *P. densiflora* extracts exert their antibacterial activities attacking the membrane, thereby increasing membrane fluidity. Membrane fluidity increase was more evident in case of Gram-negative *E. coli*. Phytochemical possessing phenolic radicals are generally reported to exert their antibacterial activities against bacterial membranes through hydroxyl groups [33]. It was postulated that the disruption of lipid bilayers in bacterial membranes could result from the accumulation of hydrophobic phenolic groups in lipid bilayers. Once they were attacked, the increase in membrane permeability, acceleration of the extensive leakage of intracellular constituents, and then destruction of membrane integrity would follow. In order to elucidate how the alteration of fluidity would correlate with the fatty acid composition of the membrane, the fatty acid composition of the tested bacteria under the extracts was determined using gas chromatography and mass spectrometry. Under adverse conditions, bacteria preserve the cell’s integrity and functionality by altering the cell membrane’s structure. The ratio of unsaturated fatty acids to saturated fatty acids (SFA) is the crucial factor affecting the fluidity and solidity of the membrane [34]. In general, production of branched and/or unsaturated fatty acids has a tendency to increase the membrane fluidity, whereas saturated straight chain fatty acids are more densely packed and thus resulting in the loss of fluidity [35]. With the increase in treatment time, SFAs in *E. coli* gradually increased and C18:0 was significantly higher than the control group at 6 h. Noticeably, trans C18:1n-9 was observed at 6 h and 9 h upon exposing the extract. The increase in membrane fluidity was paralleled with the decrease in SFAs in *S. aureus* under *P. densiflora* treatment suggesting that the increase in the content of SFAs may have contributed to the decrease in membrane fluidity to compensate for the increased fluidity caused by *P. densiflora* extracts. In this study, *E. coli* may have converted into trans UFAs in order to resist the increasing tendency of membrane fluidity under the *P. densiflora* extracts. It has been reported that in bacteria, the conversion of cis-UFAs to trans-UFAs was accompanied by the increase in fluidity, and this was interpreted as an adaptation to resist environmental stress [36]. The reason why *S. aureus* would show less increase in trans-UFAs is unclear at present. The current results of fluorescence polarization analysis and the fatty acid composition changes as determined with gas chromatography mass spectrometry suggest that *P. densiflora* caused the increase in membrane fluidity, and this alteration may have led to the fatty acid composition changes building more saturated fatty acids. It is believed that the prominent and major mechanism of the antibacterial activities of plant origin compounds involves the membrane fluidity alteration, however, other kinds of mechanisms have not been thoroughly investigated. Therefore, further examination of the control of fatty acid biosynthetic genes by *P. densiflora* would provide deeper insights into the mechanisms. Realtime q-PCR analysis showed that *P. densiflora* extracts induced a down-regulation of most of the fatty acid biosynthesis-associated genes in both *E. coli* and *S. aureus* at 6 h. However, when the incubation time was extended to 9 and 12 h, this effect disappeared. The expression levels of the *fabI* gene, known for the rate-limiting step of fatty acid biosynthesis, were decreased at 9 h in *E. coli*. Overall, the extracts appeared to have a less prominent effect on fatty acid synthetic genes in the case of *E. coli* and the stronger stimulating effects in *S aureus*.

The global regulators of stress response, *rpoS* (*E. coli*) and *sigB* (*S. aureus*), were also examined under the influence of the extracts. The transcription levels of *rpoS* decreased at 6 h, unlike *sigB*. These two stress responsive genes followed similar patterns of FA biosynthesis genes under the extract treatment. In *E. coli*, the *rpoS* gene has been indicated as a major factor in inducing *cfa* gene, and thus *cfa* as well as *fabG*, *fabI*, and *fabD* may preside in the same operons, unlike *fabA* gene. Under stress conditions, *rpoS* gene appeared to play a significant role in expression of an array of genes in entering the stationary phase under stress conditions. *SigB* gene expression is increased under stress conditions to overcome growth restriction and may control a group of genes related to stress response. The results of this study revealed how the principal mechanism of antibacterial activities of *P. densiflora* extract involved the increase in membrane fluidity in both of the Gram-positive and Gram-negative bacteria studied, and the membrane fatty acids were shifted towards higher proportions of saturated fatty acids in a greater degree in case of Gram-positive bacteria. There were also differences between Gram-positive and Gram–negative bacteria in the response to the extracts in the genes expressed showing greater alteration of fatty acid synthesis genes as well as stress responsive genes. Overall, these findings suggest that Gram-positive bacteria are better targets of germ control because of these patterns of responses. From this study, it seems important to utilize the capacity of Staphylococcus aureus self-resisting mechanisms in regulation of fatty acid biosynthesis genes to exert antibacterial functions. The detailed relationship between gene regulation and antibacterial activities needs further molecular mechanistic studies. Also, for the further mechanistic studies and validation of the targets of naturally active ingredients, it seems necessary to apply the recently described techniques including single-cell multiomics [37]. Single-cell multiomics technology has an advantage of using a single cell instead of tissue or organs in discovering the precise molecular mechanisms of many traditional medicinal plants including pine needles. A proper understanding of the mechanisms of antibacterial activities of plan-origin extracts is of great importance to bring about more appropriate applications of antimicrobial plant extracts as potential alternative food preservatives in the food industry.

## 4. Materials and Methods

### 4.1. Sample Preparation

**Figure 10:**
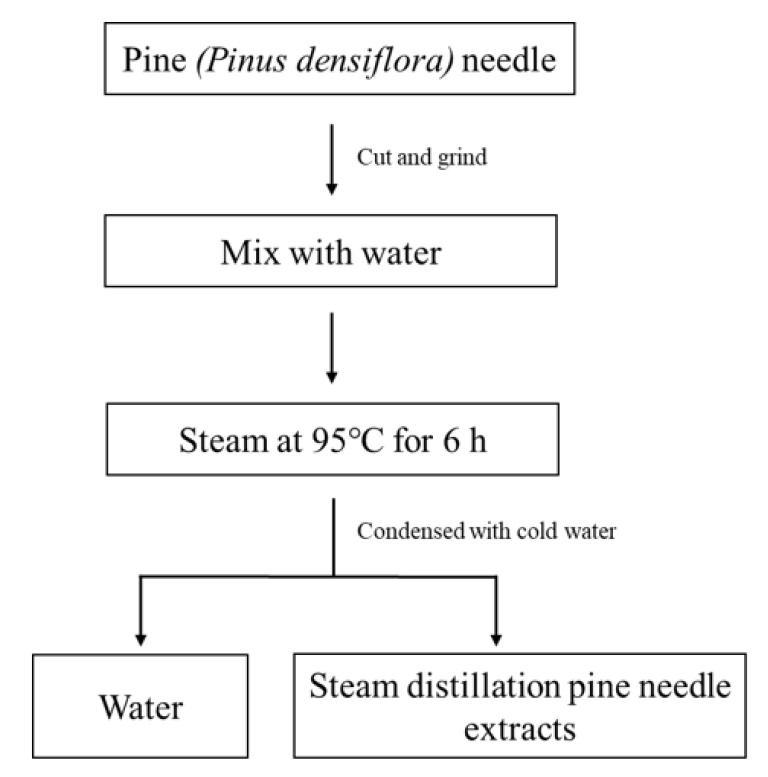
The extraction process to prepare the steam distilled pine needle (*Pinus densiflora*) extracts.

Steam distilled pine needle extracts (SDPNE) were provided by KCWELL (KCWELL Co., Ltd., Seoul, Republic of Korea). The SDPNE was prepared simply through the following process (Figure 10). The washed *P. densiflora* needle was cut to 10 mm and then ground to 3 mm. After that, 230 kg of water was added to 150 kg of ground pine needle. Then, steam was applied at 95 °C for 6 h. The vaporized water and essential oil were condensed with cold water, and then the water and essential oil were separated to collect the supernatant.

### 4.2. Total Phenolic Content

The total phenolic content (TPC) was determined according to the method described by Folin and Denis (1912) [38] with minor modifications. An aliquot of 0.2 mL of 2N Folin-Ciocalteu’s reagent was mixed in 0.2 mL of diluted sample (1:9 diluted by distilled water). After 3 min, 0.4 mL of 10% sodium carbonate solution (Na_2_CO_3_) was added and incubated at room temperature in the dark for 1 h. Absorbance was measured at 725 nm using an ELISA microplate reader (Thermo Fisher Scientific, Waltham, MA, USA). The TPC in the sample was expressed as mg of gallic acid equivalents per gram extracts (mg GAE/g).

### 4.3. Total Flavonoid Content

The total flavonoid content (TFC) was determined according to the method described by Moreno [39] with minor modifications. An aliquot of 0.1 mL of SDPNE was added to the test tube and mixed with 0.02 mL of 10% aluminum nitrate [Al (NO_3_)_3_·9H_2_O) and 0.02 mL of 1 M potassium acetate (CH_3_COOK). After adding 0.86 mL of ethyl alcohol, a solution was reacted for 30 min at room temperature in the dark. The absorbance of the sample solution was measured at 415 nm. The TFC in the sample was expressed as mg of quercetin equivalents per 100 g (mg quercetin equivalents/100 g samples).

### 4.4. 2,2′-Azino-bis(3-ethylbenzthiazoline-6-sulfonic acid) (ABTS) Radical Scavenging Activity

ABTS (2,2′-azino-bis(3-ethylbenzthiazoline-6-sulfonic acid)) radical scavenging assay was performed to determine the antioxidant activity of SDPNE following method [40] with minor modifications. ABTS reagent was prepared by mixing ABTS and potassium persulfate (K_2_S_2_O_8_) stock solutions in a 1:1 (*v*/*v*) ratio and was stored in the dark for 16–24 h at room temperature. Before use, the ABTS solution was diluted to maintain an absorbance range of 0.7–0.9 at 734 nm. Different concentrations (0.1 and 1%) of SDPNE were prepared by dilution with 95% ethyl alcohol. The ABTS reagent was added to each well with sample and incubated at room temperature in the dark. The absorbance was measured at 734 nm using an ELISA microplate reader. Trolox (250 μM) or L-ascorbic acid (1 mM) were used as positive controls, and every analysis was performed in triplicate. The ABTS radical scavenging activity of the sample was calculated as:

ABTS (%) = [(Control O.D. − Sample O.D.)/Control O.D.] × 100

### 4.5. Ferric Reducing Antioxidant Power (FRAP) Assay

FRAP was measured using the Benzie and Strain’s method [41] with minor modification. FRAP solution was prepared by mixing all three sub-solutions: Sodium acetate buffer (0.3 M, pH 3.6, C_2_H_3_NaO_2_·3H_2_O), 10 mM TPTZ (2,4,6-tripyridyl-s-triazine) in 40 mM HCl, and 20 mM FeCl_3_·6H_2_O in a 10:1:1 ratio (*v*/*v*/*v*). SDPNE was diluted with 95% ethyl alcohol into different concentrations (0.1 and 1%). After incubation at 37 °C for 15 min, 180 μL of FRAP reagent and 20 μL of the sample were added to each well of 96 well plates and reacted at room temperature for 30 min. The absorbance was then measured at 595 nm using an ELISA microplate reader and was performed in triplicate. Trolox (250 μM) was used as a positive control. The iron (II) sulfate heptahydrate (FeSO_4_·7H_2_O) was used as the standard to create the calibration curve, and FRAP results were expressed as FeSO_4_·7H_2_O equivalents (μM).

### 4.6. Microorganisms and Materials

The microorganisms used in the antibacterial activity test were from the Korean Collection for Type Cultures (KCTC) and the American Type Culture Collection (ATCC). The medium used for *Staphylococcus aureus* (*S. aureus*, Culture No. ATCC 25923)*, Salmonella enteritidis* (*S. enteritidis*, Culture No. ATCC 13076), and *Bacillus subtilis* (*B. subtilis*, Culture No. KCTC 1021) were Tryptic Soy Agar (TSA, Oxoid Ltd., Hants, UK) and Tryptic Soy Broth (TSB, Oxoid Ltd., Hants, UK). Nutrient Agar (NA, Difco, MI, USA) and Broth Nutrient (NB, Difco, MI, USA) were used for *Escherichia coli* (*E. coli*, Culture No. KCTC 2441). Brain Heart Infusion (BHI, Difco, MI, USA) and Brain Heart Infusion Agar (BHIA, Difco, MI, USA) were used for *Listeria monocytogenes* (*L. monocytogenes*, Culture No. ATCC 19115).

### 4.7. Antibacterial Activity

The antibacterial activity of SDPNE was performed using the paper disk method [42]. All the bacterial cultures (sub-cultured before assay) were diluted with sterile water to obtain a bacterial suspension of OD_600nm_ = 0.2~0.3. Petri dish (Ø6 mm, ADVANTEC, Saijo, Japan) containing 20 mL of NA medium and TSA medium were inoculated with 0.1 mL of bacterial suspension and dried in a sterile chamber. The paper disk was impregnated with 20 μL of SDPNE with different concentrations (100, 75, 50, 25, and 10%), placed on the inoculated medium, and cultured at 37 °C for 24 and 48 h. The sterile water was also used as the negative control. All the experiments were performed in triplicate.

### 4.8. Measurement of Bacterial Growth

To determine the effect of SDPNE on the growth behaviors of *E. coli* and *S. aureus*, bacterial growth was monitored. Pre-cultured *E. coli* and *S. aureus* cells were transferred to a fresh NB or TSB liquid medium (50 mL, OD_600_ ≈ 0.08) and supplemented with different SDPNE or equal volumes of NB or TSB (control). The cells were further incubated (200 rpm, 37 °C), and cell growth was monitored every 3 h by recording OD_600_ using the Shimadzu UV-1800 spectrophotometer. The growth rate (μ) of *E. coli* and *S. aureus* in the presence of various concentrations of SDPNE was calculated using OD_600_ values according to the following equation:

μ = (ln *A*_1_ − ln *A*_2_)/(*t*_1_ − *t*_2_)

*A*_1_ and *A*_2_ are the OD_600_ values at the culture times *t*_1_ and *t*_2_, respectively.

### 4.9. Membrane Fluidity Analysis

The effects of 0.1% SDPNE on *E. coli* and *S. aureus* were observed at 6 h, 9 h, and 12 h. The bacterial cultures were centrifuged at 5000× *g* for 10 min at 25 °C and washed twice with phosphate-buffered saline (PBS, pH 7.2). The harvested bacterial cells were used to analyze membrane fluidity, FAs composition, and transcription level. In the present work, the pellet was resuspended in the same buffer (NB or TSB) to 0.5 at OD_600nm_, followed by the addition of 2.0 mM of the fluorescent membrane probe, 1,6-diphenyl-1,3,5-hexatriene (DPH, Sigma-Aldrich, St. Louis, MO, USA) dissolved in tetrahydrofuran to a final concentration of 2.0 μM. After incubation at 37 °C for 1 h in the dark, the samples were centrifuged and resuspended in the same volume of PBS buffer. The cell suspensions were immediately used for fluorescence polarization analysis by a Synergy HTX (BioTek Instruments, Winooski, VT, USA), using excitation and emission wavelengths of 360 and 430 nm (5.0/5.0 nm slit widths), respectively. Fluorescence polarization was calculated as follows:

P = (*I_vv_* − *GI_vv_*)/(*I_vv_* + *GI_vh_*)

where P is fluorescence polarization, *G* is the instrument grating factor, *I_vh_* denotes the intensity of the emitted beam in the horizontal direction, and *I_vv_* is the intensity of the emitted beam in the vertical direction.

### 4.10. Membrane Lipid Extraction

After freeze drying, the Ministry of Food and Drug Safety’s (2022) method was employed to extract lipids from harvested bacterial pellets. In total, 50 mg of Pyrogallol and 1 mL of Triundecanoin (Larodan, Solna, Stockholm County, Sweden) were added to the dry biomass (100 mg). When boiling, 1 mL of ethanol and HCl (8.3 M, 5 mL) were added for mixing. The tubes were sealed and placed in a water bath at 70–80 °C for stirring once every 10 min and this was repeated four times. After cooling to room temperature, ethanol (2 mL) and diethyl ether (12.5 mL, J.T. Baker, Phillipsburg, NJ, USA) were mixed and shaken for 5 min. Petroleum ether (12.5 mL, Sigma Aldrich, MA, USA) was added and shaken for 5 min. The mixture was centrifuged at 50× *g* for 5 min, and the supernatant was extracted and volatilized in nitrogen. Fatty acid methyl esters were analyzed using a gas chromatography-mass spectrometer (GC-MS).

### 4.11. Total RNA and Gene Expression Analysis (Realtime qRT-PCR)

The total RNA in the samples was extracted with TRIzol™ Reagent (Invitrogen, Carlsbad, CA, USA) following the manufacturer’s instructions. The concentration and purity of the RNA were determined by measuring OD_260_ and OD_280_ using a NanoDrop one UV-Vis Spectrophotometer (Thermo Fisher Scientific, Waltham, MA, USA). A total of 1 µg RNA, treated with 50 μM Oligo d(T)_20_ primer and 10 mM dNTP to avoid DNA contamination, was reverse-transcribed into cDNA. Thereafter, the cDNAs were used for realtime qRT-PCR analyses using 10 × PCR buffer, Taq DNA polymerase, SYBR Green, 20 mM MgCl_2_, and 2.5 mM dNTP which were mixed. qRT-PCR analysis was performed using the CFX96^TM^ Touch Realtime PCR Detection System (Bio-Rad, Hercules, CA, USA). *Gyrb* and 16S rRNA were used as an internal control, and the relative gene expression level was calculated by the 2^−∆∆CT^ method (comparative CT method) (Livak and Schmittgen, 2001). The primer sets for the target gene expressions are listed in Table 4.

### 4.12. Statistical Analysis

SPSS (ver. 24.0, SPSS Inc., Chicago, IL, USA) was used to verify the statistical significance of the three independent replicates’ data, which were expressed as mean ± standard deviation. All data were calculated by one-way analysis of variance (one-way ANOVA), followed by Duncan’s multiple range test. Statistical significance was defined as a *p*-value of less than 0.05.

## Figures and Tables

**Table 1:** Total phenolic and total flavonoid contents in steam distilled pine needle extracts.

Components	Steam Distillation
Total phenolic content (mg GAE/g ^(1)^)	3.610 ± 0.002
Total flavonoid content (mg QE/100 g ^(2)^)	24.970 ± 1.550

Values are presented as means ± standard deviation. ^(1)^ mg gallic acid equivalents per gram. ^(2)^ mg quercetin equivalents per 100 g.

**Table 2:** Membrane fatty acid composition of ratio of saturated fatty acids to unsaturated fatty acids expressed to steam distilled pine needle extracts at a concentration of 0.1% at 6, 9, and 12 h.

Bacteria	Fatty Acids	Fatty Acid Composition (%)					
		6 h		9 h		12 h	
		CG ^(1)^	0.1% SDPNE	CG ^(1)^	0.1% SDPNE	CG ^(1)^	0.1% SDPNE
*E. coli*	SFAs ^(2)^	55.94	57.37	57.43	58.14	60.71	61.19
	UFAs ^(2)^	44.06	42.63	42.57	41.86	39.29	38.81
	Ratio ^(3)^	1.27:1	1.35:1	1.35:1	1.39:1	1.55:1	1.58:1
*S. aureus*	SFAs ^(2)^	59.54	69.77	53.96	61.31	54.02	59.21
	UFAs ^(2)^	40.46	30.23	46.04	38.69	45.98	40.79
	Ratio ^(3)^	1.47:1	2.31:1	1.17:1	1.58:1	1.18:1	1.45:1

^(1)^ Control group. ^(2)^ SFA (saturated fatty acids) UFA (unsaturated fatty acids). ^(3)^ Ratio of saturated fatty acids to unsaturated fatty acids.

**Table 3:** Membrane fatty acid composition of *Escherichia coli* and *Staphylococcus aureus* at different time points after treatment with 0.1% SDPNE.

Bacteria	Fatty Acids	Fatty Acid Composition (%)					
		6 h		9 h		12 h	
		CG ^(1)^	0.1% SDPNE	CG ^(1)^	0.1% SDPNE	CG ^(1)^	0.1% SDPNE
*E. coli*	C12:0	2.82	3.23	2.94	3.10	3.10	3.25
	C14:0	7.85	8.53	7.89	7.49	7.86	8.03
	C15:0	1.01	1.38	1.28	-	0.95	0.76
	C16:0	40.64	39.63	41.65	42.38	44.29	45.12
	C17:0	1.21	1.15	1.10	1.55	1.90	1.15
	C18:0	2.41	3.46	2.57	3.62	2.62	2.87
	C16:1	12.88	13.13	12.29	8.53	9.05	8.41
	C20:1	-	-	-	-	0.71	0.57
	*Cis*-C18:1n-9	28.77	25.35	27.89	27.13	25.95	26.39
	*Trans*-C18:1n-9	-	2.30	-	2.07	-	-
	Isomerization ^(2)^	-	8.33	-	7.08	-	-
	*Cis*-C18:2n-6	0.80	-	0.73	1.55	1.19	1.34
	*Trans*-C18:2n-6	1.61	1.84	1.65	2.58	2.38	2.10
	Isomerization ^(2)^	66.67	100.00	69.23	62.50	66.67	61.11
*S. aureus*	C14:0	2.31	2.33	-	2.38	-	1.97
	C16:0	5.20	5.81	5.04	4.76	6.32	6.58
	C18:0	21.97	24.42	20.86	21.43	21.84	21.71
	C20:0	30.06	37.21	28.06	32.74	25.86	28.95
	C18:3n-6	2.31	-	-	-	-	-
	C20:1	2.89	2.91	2.88	2.98	4.02	2.63
	*Cis*-C18:1n-9	11.56	10.47	12.95	11.90	11.49	11.84
	*Trans*-C18:1n-9	21.39	13.95	28.06	21.43	27.01	23.03
	Isomerization ^(2)^	35.09	42.86	31.58	35.71	29.85	33.96
	*Cis*-C18:2n-6	-	-	-	-	-	-
	*Trans*-C18:2n-6	2.31	2.91	2.16	2.38	3.45	3.29

^(1)^ Control group. ^(2)^ The percentage of *trans*- relative to the total amount of *cis*- and *trans*-.

**Table 4:** Primer sets for gene expression studies.

Bacteria	Target	Primer (5′→3′)
*E. coli*	*fabA*	Fw—CCAGGAACGTATCGCACAAG Rv—CGCTGAACAAGTCCGATCAGT
	*fabD*	Fw—CGTTGGAATGCTGGCTGATA Rv—CTGCTGCCATACGCGATACA
	*fabG*	Fw—CGCTCAGGCGATCAGTGATT Rv—ACCGGCATTATTGACCAGGA
	*fabI*	Fw—CGGTAAGCGCATTCTGGTAA Rv—TGCTGGCATCTTCTGCAACA
	*cfa*	Fw—CTTAGCCGTGCCGGTATAGC Rv—CGAGACCTGCGCGTAAGACT
	*rpos*	Fw—CGCCGGATGATCGAGAGTAA Rv—CCACCAGGTTGCGTATGTTG
	*gyrB*	Fw—TGCGTGGCTTGCTGGAAGAA Rv—CGGGTTCATCTCGCCCAGAC
*S. aureus*	*fabD*	Fw—TTGACGCATAGTTCGGCATT Rv—ACTGCAGCCATGCTTCCTACA
	*fabF*	Fw—GACGTGTGAGTCCATTTTTC Rv—ACCACCAGTAATCATTGCAT
	*fabG*	Fw—GTTGCCGATGCTGATGAAGT Rv—TCATCCCACTCTTGTTCTTTCA
	*fabH*	Fw—GATAACCGCACCTGCACCAT Rv—TGGATCAACTTGCAGCATGTT
	*fabI*	Fw—GAAGACTTACGCGGACGCTT Rv—TGCTACCACCTTCTGGCATTA
	*sigB*	Fw—TGAAGATGCCAAGATTGCAGT Rv—CTAGGCCACCTTCGCGTAA
	*16s RNA*	Fw—CGGTGAATACGTTCYCGG Rv—GGWTACCTTGTTACGACTT

## Data Availability

Data are included in the article.
